# Comparative Analysis of Junior and Senior Clinician Educator Evaluation of Relevant Articles Within Medical Education

**DOI:** 10.7759/cureus.2594

**Published:** 2018-05-08

**Authors:** Michael Gottlieb, Kevin Lam, Saif Shamshoon, Teresa M Chan

**Affiliations:** 1 Department of Emergency Medicine, Rush University Medical Center; 2 School of Medicine, McMaster University, Hamilton, CAN; 3 Faculty of Health Sciences, Department of Medicine, Division of Emergency Medicine, McMaster University, Hamilton, CAN

**Keywords:** medical education, faculty development, junior educators, senior educators

## Abstract

Introduction

It may be difficult for junior clinician educators (JCEs) to get a grasp of pertinent literature and determine which are most relevant to their learning, due to limited experience and lack of formalized system to rank all available resources with respect to their value for JCEs. Our study aimed to identify whether senior clinician educators (SCEs) and JCEs differ in their selection of what they perceive as key medical education articles.

Methods

As a part of the Academic Life in Emergency Medicine (ALiEM) Faculty Incubator program, we developed a series of primer articles for JCEs by identifying and discussing key articles within specific medical education arenas, which were designed to enhance the reader's educational growth. Each set of articles within the primer series were selected based on data collected from JCEs and SCEs, who ranked the specific articles with respect to their perceived relevancy to the JCEs. ANOVA analysis was performed for each of the series to determine whether there was a statistically significant difference between JCE and SCE rating of articles.

Results

Two-hundred-and-sixteen total articles were evaluated within the nine primer topics. No statistically significant difference was found between the rankings of papers by JCEs and SCEs (effect size: 0.06; 95% CI: -0.27 to 0.40). However, a subgroup analysis of the data found that three of the nine primers showed statistically significant divergence based on seniority (p < 0.05).

Conclusions

Based on the data, the involvement of JCEs in the consensus-building process was important in identifying divergence in views between JCEs and SCEs in one-third of cases. Our findings suggest that it is important to involve JCEs in selecting articles that are worthwhile for their learning, since SCEs may not fully understand their needs.

## Introduction

There is a vast amount of literature within medical education and selecting the most high yield resources can be challenging for many educators. As there is no formalized system to rank all available resources with respect to their value for junior clinician educators (JCEs), many JCEs rely on recommendations from mentors or other sources [1-10]. It is generally believed that experts (i.e., senior clinician educators) are the best resource given their prior experience within the field. However, JCEs can provide a different perspective as they are active learners with a better understanding of their knowledge deficits and needs. While it is generally assumed that senior clinician educators (SCEs) have a better ability to identify articles than JCEs, no prior studies have specifically evaluated this. The purpose of this study was to evaluate how JCEs differ from SCEs with respect to the selection of key medical education articles.

## Materials and methods

Ethics statement

The study was exempt from ethics approval according to the Hamilton Integrated Research Ethics Board as it involves program evaluation of clinician educators for the purposes of assessment, management and improvement purposes and is therefore exempt under the Tri-Council Policy Statement 2 (TCPS2), Article 2.5.

Population

The Academic Life in Emergency Medicine (ALiEM) Faculty Incubator program was a year-long, virtual faculty development program for JCEs. Primer articles were developed for JCEs on topics relevant to medical education [1-9]. The study selection methodology included a modified Delphi approach utilizing both JCEs (i.e., junior faculty members) and SCEs (i.e., experienced educators with >10 peer-reviewed publications within the field).

Study design

The intention of this study was to compare whether JCEs and SCEs had differences in their rating of which articles were important for JCEs to critically review. Both JCEs and SCEs reviewed all of the included articles and provided a rating for each article using a Likert scale from one (i.e., “Unimportant for Junior Faculty; Unlikely to Significantly Impact Junior Faculty”) to seven (i.e., “Essential for Junior Faculty; Illuminating; Highly Useful”). Raters were blinded to the scores of the other raters.

Outcomes

Our primary outcome was to compare the ratings between JCEs and SCEs to determine if the two groups had different opinions on which articles were essential for junior faculty to read.

Analytic plan

Data was analysed in STATA 13.0 (Stata Corp LP, College Station, TX). A multilevel model regression analysis was performed to determine whether the independent variable seniority (SCE vs. JCE) affected the scoring of the importance of papers (dependent variable). We adjusted for clustering by study in our analysis. Subgroup analyses for each of the nine primer series were performed using ANOVA to determine whether there were differences in ratings between SCEs and JCEs for specific topics.

## Results

Two-hundred-and-sixteen articles were analyzed comprising 1627 total measurements. There were 33 raters, comprised of 11 SCEs and 22 JCEs. Nine total topics were evaluated. Each topic had a mean of 7.5 reviewers with a minimum of six and maximum of nine reviewers. The specific reviewers varied between topics, but there was consistently an equivalent distribution of JCEs and SCEs. There was a 100% completion rate among all reviewers. Baseline characteristics of the JCEs and SCEs are included in Table 1. Mean ratings for the JCEs and SCEs are included in Table 2.

**Table 1:** Baseline characteristics of junior and senior clinician educators.

	Junior Clinician Educator	Senior Clinician Educator
Median Years in Practice ± Interquartile Range	5.00 ± 5.75	7.00 ± 3.50
Median Number of Prior Publications ± Interquartile Range	3.50 ± 6.00	28.00 ± 14.00
Faculty Rank	Clinical Instructor: 4 (18%) Assistant Professor: 17 (77%) Associate Professor: 1 (5%) Full Professor: 0 (0%)	Clinical Instructor: 1 (9%) Assistant Professor: 5 (45%) Associate Professor: 4 (36%) Full Professor: 1 (9%)

**Table 2:** Mean rating of junior and senior clinician educators in each set of articles. SD: Standard deviation

Group	Topic	Mean Rating of Junior Clinician Educators (SD)	Mean Rating of Senior Clinician Educators (SD)	t-test
1	Education Scholarship	4.70 (1.00)	4.86 (1.06)	p = 0.18
2	Team Leadership and Collaboration	4.49 (0.97)	4.69 (0.76)	p = 0.29
3	Education Theories	4.87 (1.17)	4.65 (1.21)	p = 0.31
4	Consulting for Educators	3.90 (1.35)	5.17 (1.26)	p = 0.01
5	Teaching with Technology	4.62 (1.11)	4.51 (1.02)	p = 0.57
6	Competency-Based Medical Education	5.18 (0.93)	4.84 (1.34)	p = 0.24
7	Peer Review	4.72 (1.12)	4.61 (1.31)	p = 0.54
8	Study Design	4.94 (1.09)	4.42 (1.04)	p < 0.001
9	Program Evaluation	4.47 (1.38)	4.53 (1.21)	p = 0.72

A multilevel regression analysis was performed and demonstrated no statistically significant difference between the overall rankings of the papers by JCEs and SCEs (effect size: 0.06; 95% CI: -0.27 to 0.40; Table 3). However, a subgroup analysis of the data separated into individual topics found that three of the nine primers demonstrated a statistically significant divergence in selections by seniority (p < 0.05). These topics included education theories, consulting for educators, and study design.

**Table 3:** ANOVA results for comparing the article level ratings for junior vs. senior educators within each set of articles.

Group	Topic	Main Effect	p-value
1	Education Scholarship	F (1, 47) = 3.22	p = 0.17
2	Team Leadership and Collaboration	F (1, 33) = 2.24	p = 0.30
3	Education Theories	F (2, 101) = 4.41	p = 0.01
4	Consulting for Educators	F (1, 35) = 26.27	p < 0.001
5	Teaching with Technology	F (1, 47) = 0.25	p = 0.62
6	Competency-Based Medical Education	F (1, 41) = 0.95	p = 0.33
7	Peer Review	F (1, 47) = 0.20	p = 0.66
8	Study Design	F (1, 57) = 10.34	p = 0.0015
9	Program Evaluation	F (1, 59) = 0.09	p = 0.77
OVERALL		0.06 (-0.27, 0.40)	p = 0.712

## Discussion

Given the large number of studies published on a daily basis, it can be challenging to keep up with the medical education literature. JCEs may rely upon the recommendations of more senior clinician educators to determine which articles are most relevant. However, JCEs may be able to provide an additional perspective with a better understanding of their specific education needs. To the best of our knowledge, this is the first article evaluating for differences in article selection between JCEs and SCEs.

Overall, the data demonstrated no statistically significant difference between JCEs and SCEs with regard to article selection. This suggests that the majority of JCEs and SCEs shared similar opinions with respect to identifying articles of relevance. One potential reason for this effect is that highly valuable medical literature may be easily identifiable regardless of training due to either the importance of the specific topic or intrinsic qualities of the publication. Alternatively, it is possible that JCEs and SCEs valued the articles similarly, but for different reasons. For example, an introductory guide to scholarship in medical education may be valuable for JCEs by providing targeted advice on project development, while SCEs may value the mentorship discussion more highly [1,11].

Interestingly, a subgroup analysis demonstrated a statistically significant degree of divergence among three of the nine topics. These topics included education theories, consulting for educators, and study design. In these instances, the JCEs identified several articles that were of significant importance to their learning that were not identified by the SCEs. This is an important finding, as it demonstrates that in some cases, JCEs were able to identify additional relevant articles that were not identified by the SCEs.

It is important to consider several limitations with respect to this study. First, the study was conducted using retrospective data and is subject to all of the potential risks and biases inherent with this approach. Additionally, because there is no gold standard for determining which paper is better, it is not possible to determine with complete certainty that the articles chosen would be of significant value to all JCEs, as opposed to only those in the study group. Furthermore, the subjects selected articles from within a pre-screened group of articles and it is possible that some important articles may not have been included. However, multiple strategies were utilized to ensure that the list was as broad as possible, while still ensuring that all of the articles were relevant to the specific topic. Additionally, by providing a set list of articles, it is easier to assess for degrees of convergence and divergence between subjects. Finally, this study was conducted among a group of highly-motivated JCEs engaged in a faculty development program. It is unclear whether similar results would be found with other JCEs.

Future studies should assess which topics are most likely to have divergence, differences in rationale for article selection, and validate these findings in a larger cohort.

## Conclusions

Based on this data, the involvement of JCEs in the consensus-building process was important in identifying divergence in views between JCEs and SCEs, with significant differences noted in three of the nine topics. Our findings suggest that it is important to involve JCEs in selecting articles that are worthwhile for their learning, since SCEs may not fully identify their needs.
